# Effect of Exercises for Strengthening the Intrinsic Muscles of the Foot and Improving Ankle Mobility on Patients of Diabetic Peripheral Neuropathy

**DOI:** 10.7759/cureus.56553

**Published:** 2024-03-20

**Authors:** Daris Francis, Kotteeswaran Kandaswami, Pramod Padinhare Veedu, Alex Ponniah Subramanian

**Affiliations:** 1 Department of Physiotherapy, Saveetha College of Physiotherapy, Saveetha Institute of Medical and Technical Sciences (SIMATS), Thandalam, IND; 2 Department of Physiotherapy, Lourde Institute of Allied Health Sciences, Thaliparamba, IND; 3 Department of Medicine, Lourde Hospital, Kannur, IND

**Keywords:** paper grip test, hallux grip force, foot intrinsic muscle strengthening exercises, diabetic peripheral neuropathy, ankle dorsiflexion

## Abstract

Background and objectives

The study aimed to compare the efficacy of standard home care versus structured ankle mobility exercises in enhancing ankle and foot joint range of motion (ROM) among individuals with diabetes mellitus (DM). Additionally, it investigated the impact of foot intrinsic muscle strengthening exercises on hallux grip force in those with Diabetic Peripheral Neuropathy (DN).

Materials and methods

In a study of 200 patients with Diabetic Neuropathy (DN), selected from 345 screened diabetics with stable glucose levels and routine monitoring at a tertiary care facility, the efficacy of structured exercises versus standard care was evaluated. Participants, aged 40-70 years with mild neuropathic symptoms (neuropathy disability score of 3 to 5), were divided into two groups. Group 1 received standard care per International Diabetic Foot guidelines, while Group 2 performed targeted foot intrinsic muscle strengthening and ankle mobility exercises over eight weeks. The range of motion (ROM) for ankle and first metatarsophalangeal (MTP) joints and hallux grip force were measured, showing significant improvements in Group 2. Analysis was done using IBM SPSS.

Results

The average age of the individuals in group 1 (n=100) was 53.87±5.42 years, whereas the average age of the subjects in group 2 (n=100) was 54.23±4.69 years. The study included a total of 97 male participants, with 48 in group 1 and 49 in group 2. The groups exhibited homogeneity in terms of age, gender, duration of DM, and BMI (p>,0.05). When comparing the ROM for ankle dorsiﬂexion between the groups, it was shown that subjects in group 2 had a substantially higher ROM following exercise for both the right (27.97°±5.3° Vs 19.24°±2.54°) and left (28.55°±4.61° Vs 18.22°±1.14°) ankles compared to the patients in group 1 (p<,0.01). Nevertheless, there were statistically insignificant differences (p>,0.05) observed within the groups, both before and after the exercises, for all the variables examined except for right and left ankle dorsiflexion, and right ankle plantarflexion in group 2. Group 2 subjects exhibited a considerably greater hallux grip force compared to group 1 subjects. The mean enhanced paper grip strength for the right and left big toe of group 2 was 44±3.58 N and 43.2±2.62 N respectively. The mean enhanced paper grip force for the right and left big toe of group 1 was 38±3.11 N and 37.92±2.13 N respectively. A statistically highly significant difference was observed for hallux grip force between the groups (p<,0.01).

Conclusion

The findings of this study suggest that performing the foot intrinsic muscle strengthening and ankle mobility exercises on the foot and ankle joints can potentially enhance ROM and hallux grip force in patient groups with DN.

## Introduction

Diabetic peripheral neuropathy (DN) is observed in roughly 50% of individuals within ten years of being diagnosed with diabetic mellitus (DM). DN, infection, ischemia, and ulceration are the primary factors leading to limb loss [1,2]. Foot ulcerations represent the most expensive and crucial complications associated with DM on a global scale. Peripheral sensory neuropathy, commonly known as loss of protective sensation, predominantly impacts the lower extremities, specifically the distal parts, resulting in diabetic foot ulcers and reduces the ability to perceive pain caused by recurring trauma, which would normally serve as a protective response during walking. Additionally, diabetic persons frequently experience distinct anatomical alterations in their feet due to autonomic and motor neuropathies, intrinsic wasting of muscles, heightened metatarsal prominence, diminished joint and tendon movement, and deformities in the forefoot [3,4].

Hyperglycemia leads to the transformation of smooth tissues into a non-elastic state through the action of non-enzymatic collagenous bands. This results in stiff joints and an inflexible Achilles tendon, with the added complication of forefoot pressure exacerbating the condition due to motor impairment [1]. Sufficient ankle dorsiflexion range of motion (ROM) is essential for the proper execution of several functional tasks, including walking, jogging, and climbing stairs. Ankle dorsiflexion limitation has been identified as a contributory factor in various lower-extremity illnesses, including plantar fasciitis, ankle sprain, and patellofemoral pain syndrome [5]. Diabetic individuals may experience motor neuropathy, which can lead to muscle weakness specifically in the anterior compartment, involving the muscles in the leg and foot [6]. Hyperglycemia leads to the formation of non-enzymatic cross-links in collagen, resulting in joint stiffness and strained Achilles tendons. Consequently, the pressure on the forefoot increases due to the deterioration of motor skills [7]. These pathological abnormalities can result in restricted joint mobility and an atypical walking pattern. Patients with DM frequently exhibit a significant reduction in ankle dorsiﬂexion compared to healthy people [8].

The paper grip test (PGT) was established as a method of evaluating muscle weakness in the intrinsic muscles of the foot in individuals affected by leprosy. Inter-rater and intra-rater reliability has been demonstrated, suggesting stability in measurements over time within the same assessor. These findings underscore the potential of the PGT as a reliable tool for early identification and monitoring of muscle impairment in individuals with DM [9]. Previous studies have demonstrated that it can effectively identify muscle weakness in individuals who do not have DM [10]. Studies conducted on individuals with DM have demonstrated its potential as an effective method for the early detection of muscle deterioration [11]. The present study aimed to assess the influence of usual home care, and foot intrinsic muscle strengthening, and ankle mobility exercises, on the ROM of the ankle and other foot joints. Additionally, the study also aimed to contrast the effect of these exercises on the hallux grip force among individuals with DN.

## Materials and methods

In a prospective, randomized controlled trial involving 200 diabetic neuropathy (DN) patients, selected from 345 diabetes mellitus (DM) patients with controlled plasma glucose levels, the study was conducted at a tertiary healthcare facility. Ethical approval was obtained from the Saveetha Institute of Medical and Technical Sciences, with institutional review board number IRB 009/12/2022/IEC/SMCH. The participants, aged between 40 and 70, diagnosed with DN (neuropathy disability score of 3 to 5), both men and women with type I or type II DM, were included. They were required to demonstrate the ability to perform exercises and commit to attending the study location at least three times weekly over eight weeks. Exclusion criteria comprised patients with peripheral arterial occlusive disease, foot structural abnormalities, hyperkeratosis, ulceration history, motor impairments, retinopathy, and nephropathy.

The sample size was determined using the formula N= Z^2^p(1-p)/E^2^, with N representing sample size, Z representing the standard normal deviation at a 95% confidence level (1.96), p representing estimated prevalence, and E representing desired precision (0.05). Approval from the Institutional Ethics Committee was obtained (009/12/2022/IEC/SMCH), and eligible participants provided written informed consent. Enrollment occurred consecutively from December 1, 2022, to December 31, 2023, with 100 participants randomly assigned to each of two groups using sealed envelopes.

Group 1 received standard care per International Working Group on Diabetic Foot (IWGDF) recommendations, including screening for foot issues, maintenance of foot hygiene, moisturizing dry skin, cutting toenails straight across, and patient education to promote understanding and compliance with foot care practices.

Group 2 in the study underwent foot intrinsic muscle strengthening exercises, including short foot exercises performed in seated and standing positions with 12 repetitions per block, repeated thrice weekly for three sets, with a 2-minute interval between blocks. Additionally, they engaged in ankle mobility exercises, such as drawing the alphabet with their great toe twice daily, seated heel raises and toe raises for two sets of 10 to 15 repetitions performed two to three times daily, and calf stretching completed twice daily with two sets of 10 to 15 repetitions. These exercises aimed to enhance foot muscle strength and ankle mobility among diabetic nephropathy patients, offering a structured regimen to improve their foot health and mobility. The range of motion (ROM) for the ankle and first metatarsophalangeal (MTP) joints of both feet of each patient were assessed three times by a skilled clinician using a manual goniometer, and the mean results were documented. 

During the PGT, the examiner positions a small piece of cardboard, about the dimensions of a typical business card, beneath the hallux, specifically distal to the MTP joint. The patient was instructed to grasp it firmly with their hallux. The examiner proceeds to progressively withdraw the card as the individual resists. The participants will pass the exam if they can securely grasp the cardboard, and they will fail if they are unable to do so. This test can determine if the hallux plantar flexor strength is greater than or less than a given threshold [12]. The examiner's skill and strength, however, have a significant influence on this threshold [13]. The enhanced PGT has been developed to address this constraint by substituting the pass/fail result of the original PGT with a continuous assessment of strength [14]. This was accomplished by connecting a digital dynamometer to the card that was being pulled [13]. Hallux grip force refers to the highest net force exerted beneath the region of the hallux during the PGT [14].

The current study performed the enhanced PGT using a formerly described methodology that consisted of a series of sequential steps. Before doing the test, the participants deliberately took off their footwear and socks and positioned themselves on a stable seat without armrests. A moistened cloth was utilized to cleanse the plantar region of each hallux. During the period of skin drying, the test was elucidated according to a predetermined protocol [14]. Once the skin had become desiccated, the card was positioned beneath the hallux. Participants were directed to attempt to grasp the card before the examiner initiated the use of the dynamometer to measure the highest force applied during pulling.

The statistical analysis was conducted using IBM SPSS for Windows Version 25.0 (IBM Corp., Armonk, USA). The data was calculated using the Mean ± SD, and the quantitative variables were subjected to statistical analysis using an independent sample t-test. The gender distribution was assessed using the Chi-square test. A p-value that is either below or equal to 0.05 is deemed to be statistically significant.

## Results

Among the 200 participants, 196 individuals consistently attended all the subsequent sessions. The average age of the individuals in group 1 was 53.87±5.42 years, whereas the average age of the subjects in group 2 was 54.23±4.69 years. The study included a total of 97 male participants, with 48 in group 1 and 49 in group 2. The average duration of the DM in groups 1 and 2 was 10.21±4.12 years and 11.14±4.98 years, respectively. In group 1, the individuals' BMI was 32.47±3.75 kg/m2, while in group 2, it was 33.23±3.88 kg/m2. The groups exhibited homogeneity in terms of age, gender, duration of DM, and BMI (p>0.05) (Table 1).

**Table 1:** Demographic characteristics of the study sample SD: Standard deviation; DM: diabetes mellitus

Variables	Group 1 (mean±SD)	Group 2 (mean±SD)	t-Statistic	p-value
Age (years)	53.87±5.42	54.23±4.69	-0.50	0.62
Gender (M/F)	48/52	49/51	0.18	0.67
Duration of DM (years)	10.21±4.12	11.14±4.98	1.44	0.15
BMI (Kg/m^2^)	32.47±3.75	33.23±3.88	0.44	0.66

When comparing the ROM for ankle dorsiﬂexion between the groups, it was shown that subjects in group 2 had a substantially higher ROM following exercise for both the right (27.97°±5.3°​​​​​​​ Vs 19.24°​​​​​​​±2.54°​​​​​​​) and left (28.55°​​​​​​​±4.61°​​​​​​​ Vs 18.22°​​​​​​​±1.14°​​​​​​​) ankles compared to the patients in group 1 (p<0.01). Nevertheless, there were statistically insignificant differences (p>0.05) observed within the groups, both before and after the exercises, for all the variables examined except for right and left ankle dorsiflexion, and right ankle plantarflexion in group 2 (Table 2).

**Table 2:** Comparisons of ROM of the ankle and MTP before and after the usual home care and muscle-strengthening exercise program Measurements in degrees. SD: Standard deviation; ROM: range of motion; MTP: metatarsophalangeal joint

Area	Before exercise (mean±SD)	After exercise (mean±SD)	t-Statistics	p-value
Right ankle dorsiflexion				
Group 1	18.23±6.31	19.24±2.54	-1.48	0.14
Group 2	19.11±4.54	27.97±5.3	-12.7	<0.01
t-Statistics	-1.13	14.85		
p-value	0.26	<0.01		
Left ankle dorsiflexion				
Group 1	17.3±6.2	18.22±1.14	-1.46	0.15
Group 2	17.47±4.1	28.55±4.61	-17.96	<0.01
t-Statistics	-0.23	21.75		
p-value	0.82	<0.01		
Right ankle plantar flexion				
Group 1	30.42±5.12	31.84±5.2	-1.95	0.053
Group 2	29.73±5.84	31.27±4.11	2.16	0.03
t-Statistics	0.9	-0.86		
p-value	0.37	0.39		
Left ankle plantar flexion				
Group 1	30.7±6.8	31.42±6.02	-0.79	0.43
Group 2	31.34±5.3	32.07±6.14	0.9	0.37
t-Statistics	-0.74	0.76		
p-value	0.46	0.45		
Right first MTP flexion				
Group 1	33.58±5.45	34.66±5.02	-1.46	0.15
Group 2	34.07±4.17	35.19±5.5	1.62	0.11
t-Statistics	-0.71	0.71		
p-value	0.48	0.48		
Left first MTP flexion				
Group 1	35.78±7.27	37.3±4.1	-1.82	0.07
Group 2	36.01±6.3	36.98±3.2	1.37	0.17
t-Statistics	-0.24	-0.62		
p-value	0.81	0.54		
Right first MTP extension				
Group 1	37.8±6.33	39.27±5.77	-1.72	0.09
Group 2	37.49±5.77	38.66±5.5	-0.77	0.45
t-Statistics	0.36	-0.77		
p-value	0.72	0.45		
Left first MTP extension				
Group 1	36.06±5.9	37.34±6.14	-1.5	0.13
Group 2	36.74±5.7	37.83±6.22	1.29	0.2
t-Statistics	-0.83	0.56		
p-value	0.41	0.58		

Both groups had comparable mean age, duration of diabetes, and BMI scores (p>0.05). Group 2 subjects exhibited a considerably greater hallux grip force compared to group 1 subjects. The mean enhanced paper grip strength for the right and left big toe of group 2 was 44±3.58 N and 43.2±2.62 N, respectively. The mean enhanced paper grip force for the right and left big toe of group 1 was 38±3.11 N and 37.92±2.13 N, respectively. A statistically highly significant difference was observed for hallux grip force between the groups (p<0.01), as presented in Table 3.

**Table 3:** Comparison of intrinsic muscle strength of the foot using paper grip test before and after the exercise program between the two groups SD: Standard deviation

Variable	Right Hallux grip force (N)	Left Hallux grip force (N)
Group 1 (mean±SD)	38±3.11	37.92±2.13
Group 2 (mean±SD)	44±3.58	43.2±2.62
t-statistics	12.65	15.64
p-value	<0.01	<0.01

## Discussion

DM is a lifetime condition, and once neuropathy and structural foot abnormalities emerge, diabetic foot cannot be reversed. Implementing preventive measures to reduce the risk of diabetic foot complications was determined to be a cost-effective strategy [1]. Salsich et al. [15] found that individuals with DN had a restricted ROM in dorsiflexion compared to individuals in the control group. This was evident in the Kin-Com maximal dorsiflexion angle, which measured 10.8°±5.2° for the DN group, and 17.6°±4.0° for the comparison group. The difference was statistically significant (p<0.001). This finding corroborates our observation of reduced dorsiflexion ROM, as quantified using a goniometer. The observed results may be attributed to the reduced ability of patients with DN to exert muscular force and active torque production, which is commonly linked with motor neuropathy. The decrease in flexibility of the foot joints due to tissue stiffness impacting the joints, along with the decrease in strength and muscle activation, are connected to an upsurge in metatarsal load [16].

In a previous study, it was found that DM patients with neuropathy had significantly reduced left ankle plantar flexion and dorsiflexion relative to non-neuropathic patients. The observed rise in ROM following a four-week home exercise program in a similar prior study suggested that joint restrictions, with or without neuropathy, caused by the inherent weakening of the muscles and tension of the Achilles tendon, can potentially be ameliorated with strengthening exercises [1]. Further, Goldsmith et al. assessed the effectiveness of a home training program for foot joints in DM patients, which included both active and passive ROM exercises. After one month of starting the program, the examination of the exercise group revealed a statistically significant decrease in joint stiffness among the participants receiving treatment [17]. Similarly, the findings of the current study suggest that the observed variations can be attributed to our workout regimen, which included not only muscle-strengthening exercises but also a combination of ROM and stretching exercises. This combination demonstrated a statistically significant variance in ankle ROMs (p<0.01)

The short foot exercises are a well-known exercise that focuses on developing the intrinsic foot muscles. It entails activating these muscles to move the first MTP joint towards the calcaneus and elevate the medial longitudinal arch, all while keeping the toes unflexed [18,19]. A previous study found that the level of activation in the abductor hallucis muscle was considerably greater in individuals conducting the short foot exercise compared to those doing the typical toe curl exercise. Therefore, these exercises are believed to be an effective method for strengthening the intrinsic foot muscles [20]. However, because of its relatively recent popularity of less than 10 years, Haun et al. have highlighted that the scarcity of studies and evidence has heightened the ambiguity surrounding the advantages of these exercises [21]. However, Huang et al. documented that short foot exercises may offer more advantages and provide an active kind of support compared to other interventions due to their impact on foot alignment [22].

In a recent study conducted by Healy et al. [12], it was confirmed that PGT can be used as an accurate indicator of muscle strength in individuals with DN. The study also highlighted that PGT provides an indirect evaluation of the force exerted by a person's grip during testing, specifically the force exerted by the hallux. While this method may accurately determine if the strength of the hallux plantar flexion muscles is greater or lesser than a certain threshold, the specific level itself seems to vary according to the examiner's approach and expertise [12]. According to Chatzistergos et al., the strong correlation between the force exerted by the big toe and the instability of one's posture indicates that it can be used as a screening tool for detecting individuals with DN who are experiencing a decline in their balance control and are prone to falling [14]. Nevertheless, given the significance of the hallux in walking and its function in preserving equilibrium [16], these findings suggest that the PGT may serve as a tool for evaluating muscle deterioration as a component of a fall risk evaluation [23].

The evidence also supports the potential usefulness of the enhanced PGT in assessing the risk of falling. It emphasizes that lower-limb muscular weakness and measurements of hallux plantar-flexion strength are key predictors in assessing muscular strength [10]. Soma et al. [24] conducted a study to assess the strength of the toe grip and the muscle function involved in toe grip exertion, with and without an ankle immobilization belt. They also examined the association between the differences in muscle activities and toe grip strength. The researchers observed that both hallux grip strength and the proportion of integrated electromyography (EMG) of the medial head of the gastrocnemius muscle were considerably higher when assessed with ankle belt immobilization as opposed to measures done without immobilization. Comparably, the findings of the current investigation emphasize variations in enhanced PGT strength among the groups, with group 2 exhibiting significantly superior performance compared to group 1 (p<0.01). Considering these variations is crucial when trying to establish a relationship between the results of the enhanced PGT and the impact of activities that strengthen the intrinsic muscles of the foot.

Menz et al. assessed the efficacy of the PGT in identifying muscular weakness [10]. The traditional PGT may possess clinical significance as a simple binary assessment of impairment in toe plantarﬂexion. The participants who did not pass the test had significantly lower strength compared to those who completed the test [10]. Hence, if an abnormal PGT indicates a decline in the foot's natural muscle function, it is necessary to conduct regular foot examinations, evaluate areas of potential high pressure, provide appropriate health education, perform weight-bearing lateral X-rays of the foot and ankle to assess any reduction in the height of the arch and provide customized footwear to prevent excessive pressure on the feet of DM patients [11].

Consequently, although the PGT is highly reliable, its efficacy as a tool to measure inherent muscle weakness is doubtful because it is likely to evaluate both inherent and external muscle strength [25]. The absence of ankle stabilization, both by direct intervention by the examiner and the use of straps to restrict ankle movement, may account for the activation of the ankle plantar flexor muscles [10] and further, the participants might have flexed their toes to grab the business card, which might have activated the long extrinsic toe flexors [26]. Also, in alternative implementations of the PGT, a maximum of three attempts per individual have been employed [9,10]. The duration of testing is another crucial factor in determining the clinical viability. Following recognized clinical guidelines, the study tested hallux grip force once for each foot, ensuring that the total testing time remained below 5 minutes [27]. The likelihood of lower limb amputation (LLA) may not be solely attributed to the patient's age, but rather to the duration of diabetes, which often correlates with age. This theory finds validation in research findings that examined the relationship between diabetes duration and diabetic foot problems (DFP), revealing a positive correlation even after adjusting for age [28]. Additional research is required to determine the minimum number of attempts necessary to accurately evaluate the strength of the hallux grip. A precise and dependable assessment of inherent muscle strength would facilitate prospective investigations in examining the causative association between inherent muscle weakness and foot/toe deformity in disorders such as DN, hallux valgus, and heel discomfort [25]. 

This study is limited by the absence of a definitive benchmark, for evaluating intrinsic weakness of the foot muscles in screening tests, against which the PGT may be assessed. The most ideal benchmark to evaluate the performance of PGT would be EMG [26]. However, logistical limitations made this impractical. It is crucial to emphasize that conclusive findings about the ability of the enhanced PGT to accurately measure and anticipate inherent muscle strength can only be obtained through prospective research. This preliminary evidence suggests that there is an obvious relationship between the value of enhanced PGT force and intrinsic muscle strength of the foot, which is an important first step towards conducting a future study on this topic.

## Conclusions

The findings of this study suggest that the hallux grip force exerted by the PGT is an indicator of the strength of the foot and ankle. This measurement could be utilized to identify any decline in muscle strength of the lower extremities. Furthermore, performing strengthening and ankle mobility exercises on the foot and ankle joints can potentially enhance ROM and hallux grip force in patient groups with DN. Irrespective of the presence of neuropathy, it is recommended that all DM patients adhere to a home exercise program for self-care in this situation.

## References

[1] Cerrahoglu L, Koşan U, Sirin TC, Ulusoy A (2016). Range of motion and plantar pressure evaluation for the effects of self-care foot exercises on diabetic patients with and without neuropathy. J Am Podiatr Med Assoc.

[2] Pecoraro RE, Reiber GE, Burgess EM (1990). Pathways to diabetic limb amputation. Basis for prevention. Diabetes Care.

[3] Barshes NR, Sigireddi M, Wrobel JS, Mahankali A, Robbins JM, Kougias P, Armstrong DG (2013). The system of care for the diabetic foot: objectives, outcomes, and opportunities. Diabet Foot Ankle.

[4] Sartor CD, Watari R, Pássaro AC, Picon AP, Hasue RH, Sacco IC (2012). Effects of a combined strengthening, stretching and functional training program versus usual-care on gait biomechanics and foot function for diabetic neuropathy: a randomized controlled trial. BMC Musculoskelet Disord.

[5] Rabin A, Kozol Z (2012). Weightbearing and nonweightbearing ankle dorsiflexion range of motion: are we measuring the same thing?. J Am Podiatr Med Assoc.

[6] Frykberg RG, Zgonis T, Armstrong DG (2006). Diabetic foot disorders: a clinical practice guideline (2006 revision). J Foot Ankle Surg.

[7] Stanley JC, Collier AM (2009). The diabetic foot and ankle. Orthop Trauma.

[8] Robinson CC, Balbinot LF, Silva MF, Achaval M, Zaro MA (2013). Plantar pressure distribution patterns of individuals with prediabetes in comparison with healthy individuals and individuals with diabetes. J Diabetes Sci Technol.

[9] de Win MM, Theuvenet WJ, Roche PW, de Bie RA, van Mameren H (2002). The paper grip test for screening on intrinsic muscle paralysis in the foot of leprosy patients. Int J Lepr Other Mycobact Dis.

[10] Menz HB, Zammit GV, Munteanu SE, Scott G (2006). Plantarflexion strength of the toes: age and gender differences and evaluation of a clinical screening test. Foot Ankle Int.

[11] Mahieu R, Coenen MN, van Bemmel T, van der Zaag-Loonen HJ, Theuvenet WJ (2016). Detecting intrinsic muscle weakness of the hallux as an addition to early-stage screening of the feet in patients with diabetes. Diabetes Res Clin Pract.

[12] Healy A, Naemi R, Sundar L, Chatzistergos P, Ramachandran A, Chockalingam N (2018). Hallux plantar flexor strength in people with diabetic neuropathy: validation of a simple clinical test. Diabetes Res Clin Pract.

[13] Mansi MK, Chockalingam N, Chatzistergos PE (2023). An exploration of the mechanistic link between the enhanced paper grip test and the risk of falling. Foot (Edinb).

[14] Chatzistergos PE, Healy A, Naemi R, Sundar L, Ramachandran A, Chockalingam N (2019). The relationship between hallux grip force and balance in people with diabetes. Gait Posture.

[15] Salsich GB, Mueller MJ, Sahrmann SA (2000). Passive ankle stiffness in subjects with diabetes and peripheral neuropathy versus an age-matched comparison group. Phys Ther.

[16] Allet L, Armand S, Golay A, Monnin D, de Bie RA, de Bruin ED (2008). Gait characteristics of diabetic patients: a systematic review. Diabetes Metab Res Rev.

[17] Goldsmith JR, Lidtke RH, Shott S (2002). The effects of range-of-motion therapy on the plantar pressures of patients with diabetes mellitus. J Am Podiatr Med Assoc.

[18] McKeon PO, Hertel J, Bramble D, Davis I (2015). The foot core system: a new paradigm for understanding intrinsic foot muscle function. Br J Sports Med.

[19] McKeon PO, Fourchet F (2015). Freeing the foot: integrating the foot core system into rehabilitation for lower extremity injuries. Clin Sports Med.

[20] Jung DY, Kim MH, Koh EK, Kwon OY, Cynn HS, Lee WH (2011). A comparison in the muscle activity of the abductor hallucis and the medial longitudinal arch angle during toe curl and short foot exercises. Phys Ther Sport.

[21] Haun C, Brown CN, Hannigan K, Johnson ST (2020). The effects of the short foot exercise on navicular drop: a critically appraised topic. J Sport Rehabil.

[22] Huang C, Chen LY, Liao YH, Masodsai K, Lin YY (2022). Effects of the short-foot exercise on foot alignment and muscle hypertrophy in flatfoot individuals: a meta-analysis. Int J Environ Res Public Health.

[23] Chou SW, Cheng HY, Chen JH, Ju YY, Lin YC, Wong MK (2009). The role of the great toe in balance performance. J Orthop Res.

[24] Soma M, Murata S, Kai Y, Nakae H, Satou Y, Murata J, Miyazaki J (2015). Comparison of toe grip strength and muscle activities during maximal toe grip strength exertion according to the presence/absence of an ankle immobilization belt. J Phys Ther Sci.

[25] Garth WP Jr, Miller ST (1989). Evaluation of claw toe deformity, weakness of the foot intrinsics, and posteromedial shin pain. Am J Sports Med.

[26] Soysa A, Hiller C, Refshauge K, Burns J (2012). Importance and challenges of measuring intrinsic foot muscle strength. J Foot Ankle Res.

[27] Thomas JI, Lane JV (2005). A pilot study to explore the predictive validity of 4 measures of falls risk in frail elderly patients. Arch Phys Med Rehabil.

[28] Nouira S, Ach T, Bellazreg F, Ben Abdelkrim A (2023). Predictive factors for lower limb amputation in type 2 diabetics. Cureus.

